# Evaluation of Classifier Performance for Multiclass Phenotype Discrimination in Untargeted Metabolomics

**DOI:** 10.3390/metabo7020030

**Published:** 2017-06-21

**Authors:** Patrick J. Trainor, Andrew P. DeFilippis, Shesh N. Rai

**Affiliations:** 1 Division of Cardiovascular Medicine, Department of Medicine, University of Louisville, 580 S. Preston St., Louisville, KY 40202, USA; andrew.defilippis@louisville.edu; 2 Department of Bioinformatics and Biostatistics, University of Louisville, 505 S. Hancock St., Louisville, KY 40202, USA; shesh.rai@louisville.edu

**Keywords:** metabolomic phenotyping, statistical classification, machine learning, discrimination, partial least squares-discriminant analysis, Random Forests, support vector machines, artificial Neural Networks, Naïve Bayes, *k*-Nearest Neighbors

## Abstract

Statistical classification is a critical component of utilizing metabolomics data for examining the molecular determinants of phenotypes. Despite this, a comprehensive and rigorous evaluation of the accuracy of classification techniques for phenotype discrimination given metabolomics data has not been conducted. We conducted such an evaluation using both simulated and real metabolomics datasets, comparing Partial Least Squares-Discriminant Analysis (PLS-DA), Sparse PLS-DA, Random Forests, Support Vector Machines (SVM), Artificial Neural Network, *k*-Nearest Neighbors (*k*-NN), and Naïve Bayes classification techniques for discrimination. We evaluated the techniques on simulated data generated to mimic global untargeted metabolomics data by incorporating realistic block-wise correlation and partial correlation structures for mimicking the correlations and metabolite clustering generated by biological processes. Over the simulation studies, covariance structures, means, and effect sizes were stochastically varied to provide consistent estimates of classifier performance over a wide range of possible scenarios. The effects of the presence of non-normal error distributions, the introduction of biological and technical outliers, unbalanced phenotype allocation, missing values due to abundances below a limit of detection, and the effect of prior-significance filtering (dimension reduction) were evaluated via simulation. In each simulation, classifier parameters, such as the number of hidden nodes in a Neural Network, were optimized by cross-validation to minimize the probability of detecting spurious results due to poorly tuned classifiers. Classifier performance was then evaluated using real metabolomics datasets of varying sample medium, sample size, and experimental design. We report that in the most realistic simulation studies that incorporated non-normal error distributions, unbalanced phenotype allocation, outliers, missing values, and dimension reduction, classifier performance (least to greatest error) was ranked as follows: SVM, Random Forest, Naïve Bayes, sPLS-DA, Neural Networks, PLS-DA and *k*-NN classifiers. When non-normal error distributions were introduced, the performance of PLS-DA and *k*-NN classifiers deteriorated further relative to the remaining techniques. Over the real datasets, a trend of better performance of SVM and Random Forest classifier performance was observed.

## 1. Introduction

As the reactants, intermediates, and products of metabolic reactions, in vivo metabolite concentrations are reflective of stable hereditary factors such as DNA sequence and epigenetic modifications as well as transient stimuli that elicit metabolic responses over varying time domains. Many diseases—including prevalent human diseases such as diabetes [1], coronary artery disease [2], heart failure [3], and cancer [4]—are either caused by or result in metabolic dysregulation. Consequently, metabolite concentrations quantified from human samples report both constitutive diseases processes such as atherosclerosis [5] and acute disease events such as myocardial infarction [6] and cerebral infarction [7]. While metabolic phenotyping is well suited to inform clinical phenotype prediction, the success of this approach depends on the discriminative power of the statistical classification techniques employed. Consequently, we sought to conduct a thorough and rigorous analysis of classifier techniques for use in metabolomics, with special attention paid to high dimensional data as a common feature of untargeted analyses. In evaluating multiple statistical classification techniques, the optimization of different objective functions will lead to different results. An objective function of maximizing biological knowledge extraction may lead to the choice of simple, interpretable classifiers. In contrast, an objective function of error minimization may lead to the selection of “black box” classification techniques such as classifier ensembles for which conducting biological inference is not straightforward. In conducting our evaluations, we have defined minimizing classification error and cross-entropy loss objective functions, predicated on the assumption that, for metabolite concentrations to inform diagnostic or prognostic predictions, accuracy is more important than model interpretability. In selecting classification techniques to evaluate, we have sought to include classifiers with widespread utilization in metabolomics (e.g., PLS-DA), ensemble methods (e.g., Random Forests), methods that allow nonlinear discrimination functions and are robust given non-normal data (e.g., Support Vector Machines and Neural Networks), and methods with embedded feature selection (e.g., Sparse PLS-DA). In order to evaluate classifier performance, we utilized simulation studies designed to emulate an analysis workflow post analytical detection and quantification of metabolite abundances—that is, we assume method-specific data processing such as peak detection, signal deconvolution, and chromatographic alignment have already been conducted. While we refer to simulated abundances as metabolites for simplicity, our evaluations would generalize to datasets with ion features that have not been grouped or annotated as compounds. In addition to simulation studies, we evaluated classifier performance across three independent clinical datasets in which a principle aim was using metabolomics to facilitate a diagnostic determination. 

We briefly introduce the classifier techniques evaluated and provide a high-level introduction to our analytical process in the following paragraphs. Partial least squares-discriminant analysis (PLS-DA) is a ubiquitous classification technique that has been widely utilized in metabolomics studies [8]. The objective of partial least squares (PLS) is to find latent components that maximize the sample covariance between sample phenotype and observed abundance data after applying linear transformations to both [9]. An advantage of PLS approaches is that the latent components are iteratively determined to maximize the remaining phenotype covariance, which facilitates straightforward dimension reduction (by considering a parsimonious set of the components that capture sufficient phenotypic variance) and can mitigate estimability issues arising from the presence of more metabolites then samples (*p* > *n*) and from multicollinearity. To generalize PLS regression to classification, a matrix of binary phenotype indicators can be used as dependent variables and a discriminant analysis such as Fisher’s discriminant analysis or nearest centroids can be conducted (hence PLS-DA). Given that metabolomics studies typically have (*p* ≫ *n*) that is, far more metabolites quantified than replicates, variable (metabolite) selection is often advisable. This artifact is especially pronounced when considering data with ion features. Sparse PLS-DA can be conceptualized as a modification of PLS-DA that embeds feature (metabolite) selection through regularization. Sparsity is enforced by penalizing the norm of the weights that define the linear transformations that relate the observed abundance data and the latent components [10,11]. Dependent on the penalization parameter, some of the individual metabolite weights may shrink to zero—effectively removing that metabolite from the model. While PLS methods aptly handle the multicollinearity present in metabolomics data due to abundance correlations within metabolic pathways, the latent components are linear combinations of the metabolites and assume metabolite abundances are approximately normally distributed. The need for nonlinear function approximation is warranted given consideration to the nonlinearity of enzyme kinetics (see, for example, [12]). Support vector machines (SVMs) are binary classifiers that seek to find linear hyperplanes that maximize the separation between classes [13]. SVMs can approximate nonlinear decision boundaries between classes by employing a linear or nonlinear mapping of the metabolite data to a higher dimensional space in which a separable or nearly-separable linear hyperplane between classes can be found. The strength of SVM classifications in nonlinear discrimination—of great benefit in metabolomics—stems from the ability of SVMs to approximate arbitrary continuous functions [14] (universal approximation). This desirable property has also been shown for Neural Networks (see, for example, [15] for a proof of universal function approximation for multilayer feedforward networks). Neural networks are so named as early work in this field [16] focused on developing mathematical models that mimic cognition—specifically, recognition via the activation of neurons and propagation of signals. A general feedforward network consists of three types of node layers: an input layer for inputting metabolite abundances, hidden layer(s) conceptualized as neurons that aggregate and process signals, and an output layer that is used for prediction (e.g., predicting phenotype). The final classification technique considered in this analysis was Random Forests. A Random Forest is an ensemble of classification or regression trees that employs bootstrap aggregation (“bagging”) and random subspace constraints to minimize the variance of model sampling [17,18]. Bagging is conducted in this context by constructing a collection of individual trees using repeated sampling with replacement from the original data and aggregating the trees into an ensemble for making predictions. Bagging is a form of model averaging that has been shown to increase accuracy in proportion to the degree to which the underlying model is sensitive to perturbations of training data [19]. Random Subspace constraints stipulate that during the iterative process of tree construction, only a random subset of metabolites will be considered in defining branch splits [20]. Enforcing a random subspace constraint improves the performance of the bagging strategy by reducing the correlation between the individual trees [18]. Naïve Bayes classifiers are derived from an application of Bayes’ Theorem to the multiclass classification problem. Naïve Bayes classifiers estimate the posterior probability of each phenotype label conditioned on observed metabolite abundances, predicated on the “naïve” assumption that the distribution of each metabolite is independent given phenotype [13,18]. The final classification technique considered, *k*-Nearest Neighbors *(k*-NN), estimates the posterior probability of each phenotype label for an observation by the empirical distribution of phenotype labels in the neighborhood of k training examples most proximal to the observation with respect to a similarity measure [21].

We chose to evaluate the performance of the selected classification techniques using simulated datasets as evaluating performance on a single or small collection of real datasets would exhibit high variance. Evaluating performance on a large number of simulated datasets allows for more precise estimates of relative performance and for directly evaluating the effects of increased noise, increased nonlinearity, and/or departures from approximate normality. A significant hurdle in simulating metabolomics data is that such data is marked by a significant degree of pairwise and higher order partial correlations [22,23]. Metabolites in the same reaction or linked reactions function as substrates, intermediates, and products thus generating complex correlation structures. As a result, simulating metabolomics data necessitates generating multivariate distributions of correlated metabolites. Furthermore, as the enzymes that catalyze biochemical reactions are often subject to regulatory processes such as feedback inhibition [12], complex partial correlation structures must also be simulated. Acknowledging this, we simulated metabolite data in blocks representing biological processes. To ensure diversity in the simulation studies, random correlation matrices were generated for each simulation. Generating random correlation matrices requires specialized methods to ensure that the resulting matrices are positive definite. For this, we employed the method developed by Lewandowski et al. [24] which generates partial correlations using a graph (network) structure known as a C-vine. In addition to simulating realistic covariance structures, to ensure simulation studies mimicked untargeted metabolomics data, biological outliers, technical outliers, and missing values arising from abundances below the limit of detection were simulated. Further details are contained in the methods section.

## 2. Results

### 2.1. Simulated Metabolomics Data

**Figure 1:**
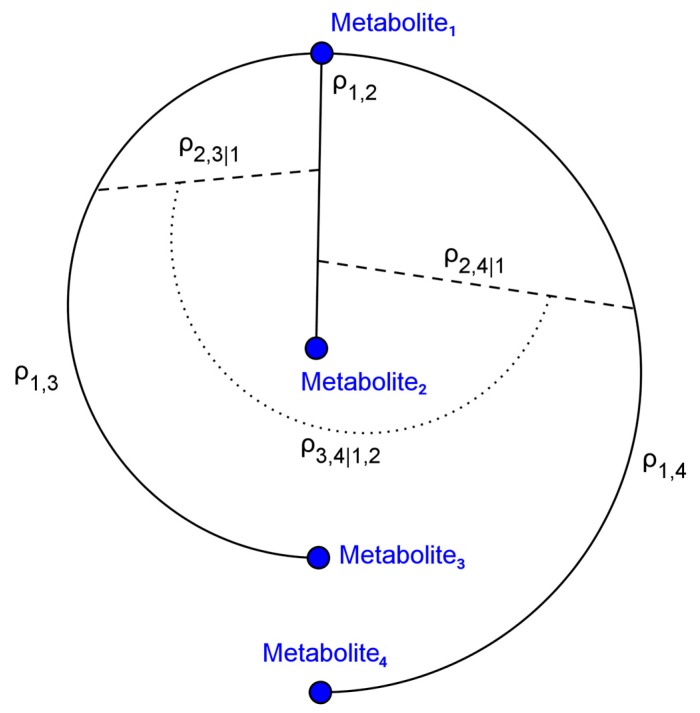
C-vine graph illustrating partial correlation structure. C-vines were utilized to generate biologically plausible metabolomics data. $\rho_{i,j}$ represents the correlation between metabolites i and j. $\rho_{i,j|k}$ represents the partial correlation between metabolites i and j after conditioning on $\rho_{k,i}$ and $\rho_{k,j}$.

For both the baseline and realistic scenarios, 1000 simulation studies were conducted. The C-vine procedure (Figure 1) for generating random covariance matrices facilitated generating clusters of simulated metabolites to mimic discrete biological processes. An important aspect of this is the generation of partial correlations, as regulatory mechanisms such as feedback inhibition may generate such relationships. 

**Figure 2:**
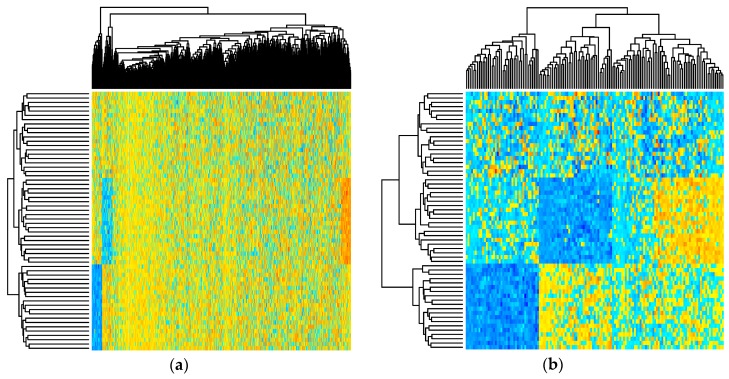
Heatmap showing simulated metabolite abundance data from a randomly selected baseline scenario before and after significance filtering. (**a**) prior to significance filtering: distinct clusters of metabolites can be discriminated as expected given the block-wise generation of correlated metabolites; (**b**) post-significance filtering.

**Figure 3:**
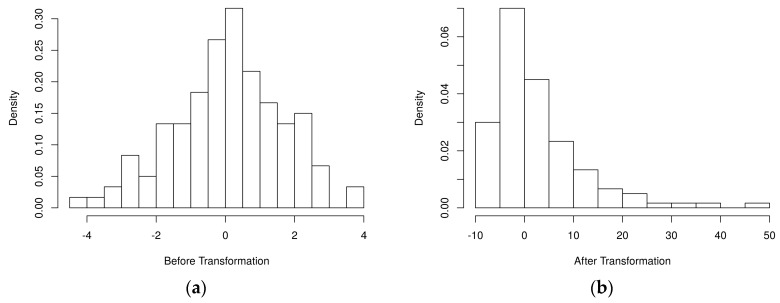
Histogram of an example simulated metabolite abundance distributions for each scenario. (**a**) baseline scenarios: metabolite abundances were simulated from multivariate normal distributions representing discrete biological processes (one metabolite shown); (**b**) realistic scenarios: metabolite abundances were initially generated as in the baseline scenarios. Then, simulated block-wise outliers were added to simulate biological outliers, metabolite-level outliers were added to simulate technical outliers, random nonlinear transformations were applied block-wise to generate non-normal error distributions, and missing values were added to simulate abundances below a limit of detection.

Figure 2 depicts the simulated metabolite abundance data from a randomly selected baseline scenario study both prior-to and post-significance filtering. Blocks of correlated metabolites are visible (column clusters) as expected given the data generation procedure. In the baseline scenarios, classifiers were evaluated on both the prior-to and post-significance filtered datasets such as those shown in Figure 2; in the realistic scenarios, further transformation was conducted. Figure 3 illustrates how abundance data was generated to follow a variety of random non-normal distributions for the realistic scenarios. In addition to introducing non-normal error distributions, in the realistic scenarios, biological and technical outliers were simulated and missing values were added to simulate abundances below a limit of detection.

### 2.2. Evaluation of Classifier Performance in Simulation Studies

#### 2.2.1. Aggregate Performance

**Figure 4:**
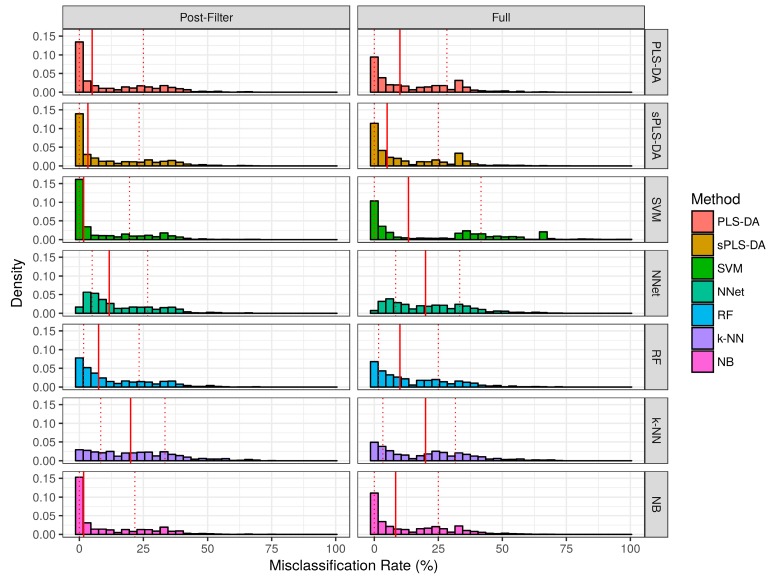
Empirical distribution of the misclassification rate observed in baseline scenario simulation studies. Solid red line represents the median of each distribution, while dashed red lines represent the 1st and 3rd quantiles (25th and 75th percentile).

The misclassification rate for each technique over the simulation studies are summarized in Figure 4 and Table 1. Over the baseline scenarios and prior-to significance filtering, sPLS-DA exhibited a lower misclassification rate than the remaining techniques (Median ± Interquartile range: 5.0% ± 25.0%). Naïve bayes classifiers demonstrated the second lowest misclassification rate (Median ± Interquartile range: 8.3% ± 25.0%) in the baseline scenarios prior to significance filtering. Following sPLS-DA and Naïve Bayes, the performance of PLS-DA, Random Forests, and SVM was similar with respect to median misclassification rate. Neural networks and *k*-NN had higher median misclassifications rate than the other techniques. Prior to significance filtering, the spread of SVM performance was greater than the remaining techniques. The application of significance filtering improved the mean and median misclassification rate for each technique. In the realistic scenarios (prior-to and post-significance filtering), the performance of PLS-DA and *k*-NN classifiers deteriorated significantly more than the other techniques. Post-significance filtering in the realistic scenarios, the ascending order of median misclassification rates was as follows: SVM, Random Forest, Naïve Bayes, sPLS-DA, Neural Networks, PLS-DA and *k*-NN classifiers.

**Figure 5:**
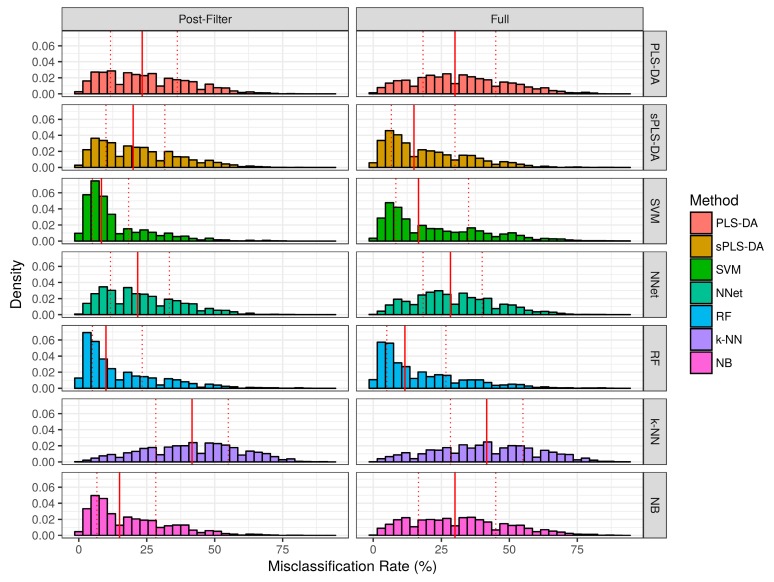
Empirical distribution of the misclassification rate observed in the realistic scenario simulation studies. The solid red line represents the median of each distribution, while dashed red lines represent the 1st and 3rd quantiles (25th and 75th percentile).

Cross-entropy loss over the simulation studies is summarized in Table 2 and Figure 5. Over the baseline scenarios prior-to significance filtering, SVM and Random Forest classifiers exhibited similar performance (Median ± IQR: 0.55 ± 0.52 and 0.70 ± 0.61, respectively); PLS-DA, sPLS-DA, Naïve Bayes, and Neural Networks were similar and higher than SVM/RF classifiers; *k*-NN classifiers exhibited the greatest cross-entropy loss. Post-significance filtering in the baseline scenarios, Naïve bayes classifiers exhibited the lowest cross-entropy loss, followed by SVM and Random Forests. As before, PLS-DA, sPLS-DA, and Neural Networks showed similar performance, while *k*-NN classifiers demonstrated the greatest cross-entropy loss. In the realistic scenarios post-significance filtering, the ascending order of cross-entropy loss was as follows: sPLS-DA, PLS-DA, Neural Networks, Random Forests, SVM, Naïve Bayes, and *k*-NN classifiers (Table 2).

#### 2.2.2. Pairwise Performance Comparisons within Simulation Studies

**Figure 6:**
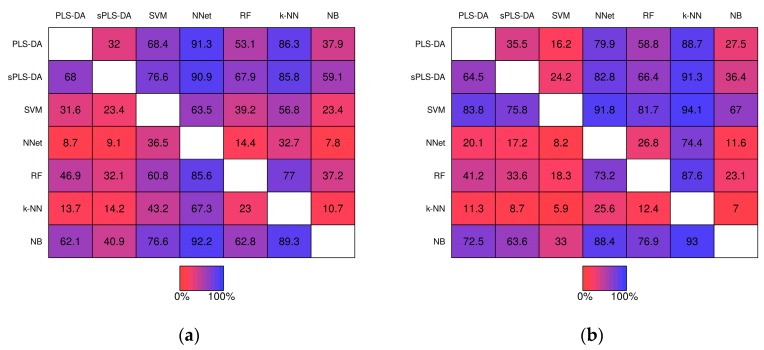

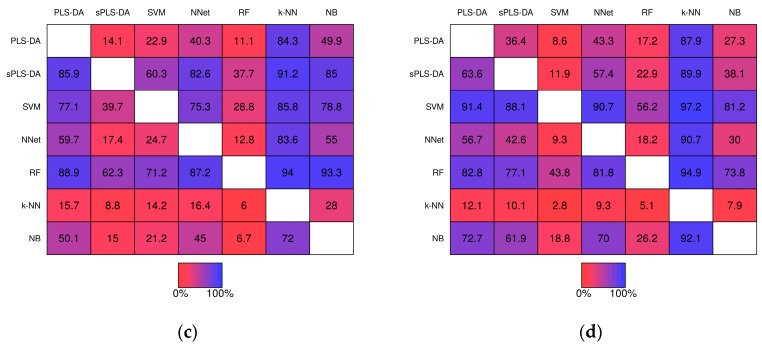
Matrices showing the proportion of the time a fixed technique performed better than another fixed technique during the same simulation study. Proportions were computed from the 1000 baseline simulation studies prior-to significance filtering (**a**) and post-significance filtering (**b**); and from the 1000 realistic simulation studies prior-to significance filtering (**c**) and post-significance filtering (**d**).

Pairwise comparisons of misclassification rate within the simulation studies are shown in Figure 6. For example, PLS-DA had a lower misclassification rate than sPLS-DA in 32.0% of the 1000 baseline scenario studies prior-to significance filtering (Figure 6a, row 1, column 2) and in 35.5% of the studies post-significance filtering (Figure 6b row 1, column 2). *k*-NN classifiers exhibited greater misclassification rate relative to the other techniques in the majority of studies with the exception of Neural Networks given baseline scenarios. In the realistic scenarios PLS-DA, exhibited a higher misclassification rate than each of the other techniques (except *k*-NN) within the same simulation the majority of the time.

### 2.3. Performance over Real Datasets

Performance over the real datasets is shown in Table 3. Over the Adenocarcinoma study data, PLS-DA, Random Forest, and Naïve Bayes observed the lowest misclassification rate (17.9%) on the test dataset prior to significance filtering. Post-significance filtering a PLS-DA classifier demonstrated the lowest misclassification (7.1%) over the test data. With respect to cross-entropy loss, Random Forests demonstrated the lowest cross-entropy loss prior to significance filtering, and an SVM classifier exhibited the lowest cross-entropy loss post-significance filtering. Over the acute myocardial infarction (MI) study data, Random Forest classifiers had the lowest misclassification rate estimated by double cross-validation prior-to and post-significance filtering (22.1% and 7.9%). With respect to cross-entropy loss, Random Forest classifiers demonstrated lowest cross-validation estimated loss, while SVM classifiers demonstrated the lowest loss following significance filtering. Finally, over the NOS1AP variants dataset, an sPLS-DA classifier demonstrated the lowest misclassification rate prior-to significance filtering (2.1%) on the test data. Post-significance filtering, a Random Forest classifier demonstrated lowest misclassification (4.2%) on the test set. Random Forest classifiers demonstrated lowest cross-entropy loss when evaluated on the test dataset prior to and post-significance filtering.

## 3. Discussion

In this report, we have detailed a rigorous and comprehensive evaluation of selected statistical classification techniques for discrimination of phenotype given metabolomic data. This work addresses a concern raised by others [8,25] that PLS-DA predominates classification in metabolomics without regard to potential limitations or misuses. In addition to PLS-DA, many of the classifier techniques included in this analysis have been utilized for achieving a classification or discrimination task in metabolomics (see for example: [26] for sPLS-DA, [27] for Random Forests, [28] for SVM, [29] for Neural Networks). Previous analyses of relative classifier performance such as a comparison of PLS-DA, SVM, and Random Forests detailed in both Gromski et al., [30] and Chen et al., [31] have been conducted over specific datasets. In the first analysis, Random Forests and SVM classifiers were shown to exhibit optimal performance and in the second the performance of Random Forests was shown to be optimal. The current study is novel in the use of simulation studies with stochastically varied parameters in order to evaluate the consistency of performance estimates in conjunction with an evaluation over a sample of real datasets. By stochastically varying parameters in the simulation studies including the number of metabolite clusters that differ between phenotypes, the effect size of differences, the degree of departure from approximate normality, the proportion of missing values, and the proportion of simulated biological and technical outliers, we have ensured that estimates of classifier performance are sufficiently general. 

A few key conclusions are supported by the analysis of misclassification rate. First, the performance of PLS-DA, Neural Networks, and *k*-nearest neighbor classifiers was generally worse than other classification techniques. The deterioration of performance of PLS-DA classifiers with the introduction of realistic metabolomics data artifacts such as non-normal error distributions, outliers, and missing values was especially pronounced. In the scenario that is likely most relevant to metabolomics practitioners (“realistic scenarios” post-significance filtering) the ordering of most accurate to least was SVM, Random Forest, Naïve Bayes, sPLS-DA, Neural Networks, PLS-DA and *k*-NN classifiers. Over these scenarios, SVM classifiers demonstrated superior performance with respect to pairwise comparisons within the same simulations, while *k*-NN demonstrated inferior performance relative to other techniques. The lackluster relative performance of *k*-NN classifiers may be attributed to needing larger sample sizes than available in the simulated and real datasets in this analysis; modified versions have been proposed previously to optimize *k*-NN for small sample size problems [32]. Another conclusion supported by this work is that regularization of PLS-DA improves accuracy in addition to encouraging a sparser classifier. This is consistent with previous work given a regression as opposed to discrimination problem. Chun and Keles [33] demonstrated that the asymptotic consistency of PLS estimators does not hold for *p* ≫ *n* and that in the regression case with *p* ≫ *n*, regularization (sPLS) substantially decreased mean square error relative to PLS.

Significant conclusions can also be drawn from the analysis of cross-entropy loss. In the scenario that is likely most relevant to metabolomics practitioners (“realistic scenarios” post-significance filtering), the cross-entropy loss ordering (least to greatest error) was sPLS-DA, PLS-DA, Neural Networks, Random Forests, SVM, Naïve Bayes, and *k*-NN classifiers. While similar cross-entropy loss performance was observed for sPLS-DA, PLS-DA, Neural Networks, Random Forests, and SVM classifiers over the realistic scenarios post significance filtering, Naïve Bayes and *k*-NN classifiers performed substantially worse. As relatively high cross-entropy loss corresponds to a relatively low predicted probability of the true phenotype, Naïve Bayes and *k*-NN classifiers have a demonstrated tendency to make such errors over simulated metabolomics data. The difference in relative performance of the classifier techniques across the two loss functions considered demonstrates the impact loss function choice on the measurement of classifier accuracy. Cross-entropy loss function has the advantage of differential penalization of phenotype predictions based on predicted phenotype probability, while 0–1 loss (yielding the misclassification rate) considers only whether the most likely phenotype label matches the true phenotype. Consequently, it may be a more appropriate measure of accuracy for probabilistic reasoning in clinical applications.

A limitation of this work is that, while we have sought to minimize the effect of algorithm parameters on observed misclassification rate and cross-entropy loss by conducting extensive parameter tuning via cross-validation, the entire parameter space was not evaluated for multiple techniques. For example, while a thorough grid search (with smoothing) was conducted to select the Gaussian kernel bandwidth parameter for the SVM classifiers, the space of kernels not evaluated remains infinite. Additionally, in the current study we have defined measures of prediction error as the objective criteria for measuring classifier performance. However, other criteria such as model interpretability may be important for practitioners. This is especially the case when classifier techniques are used for hypothesis testing or for biological inference. Additionally, throughout this analysis we have evaluated each classifier with identical preprocessing steps prior to model fitting. For example, for dimension reduction we have chosen to employ a uniform univariate significance filtering process irrespective of the classifier technique. However, there exist classifier specific methods for feature selection such as support vector machine-recursive feature Elimination (SVM-RFE) [34] that may optimize the performance of a specific technique. 

## 4. Materials and Methods 

### 4.1. Simulated Metabolomics Data

Evaluation of classifier techniques for metabolomics-based phenotype discrimination requires simulation studies that realistically mimic data captured using analytical methods such as nuclear magnetic resonance or chromatography-coupled mass spectrometry from biological samples (e.g., cell or biofluid extract). While the distribution of metabolite abundances may have platform and/or sample medium specific artifacts, we posit that six features are common to untargeted metabolomics studies: (1) significant correlations and higher-order partial correlations between metabolites within biological processes, (2) a small proportion of differentially abundant metabolites localized specific biological processes, (3) a large number of quantified metabolites relative to sample size—most demonstrating variance orthogonal to phenotype, (4) non-Gaussian error distributions and nonlinear relationships between metabolite abundances and phenotype attributes, (5) metabolite abundance levels below a limit of detection, and (6) presence of biological and technical outliers.

Metabolites within biochemical processes are related by substrate, intermediate, and product relations thus generating complex correlation structures. Consequently, we generated simulated abundance data to follow multivariate distributions with covariance structures that allow for mimicking biological processes. Further, as the enzymes that catalyze biochemical reactions are often subject to regulatory processes such as feedback inhibition [12], we simulated complex partial correlation structures. We represent metabolite abundance data as a matrix X of dimension ***n*** × ***p*** given ***n*** samples and ***p*** metabolites, sample phenotype labels as a vector **y** or as a matrix of binary indicators Y. For each simulation study dataset we generated 40 multivariate blocks of 25 metabolites. Each block, $X_{k}$ was generated such that $X_{k}$ followed a multivariate Gaussian distribution, that is: $X_{k}\sim N\left(\mu_{k},\Sigma_{k}\right)$. The covariance matrices $\Sigma_{k}$ were each randomly generated using C-vines for simulating partial correlations between metabolites [24]. The algorithm for generating correlation matrices utilizing C-vines is presented below (Algorithm 1) and was developed by Lewandowski, Kurowicka and Joe [24]: 

**Algorithm 1**1: *Initialize*
β=η+(d−1)/2 2: ***For***
k∈{1,2,…,d−1}
***do:*** 3:  β←β−1/2 4:  ***For***
i∈{k+1,k+2,…,d}
***do**:* 5:     *Generate*
$\rho_{k,i;1,2,\ldots ,k-1}\sim Beta\left(\beta ,\beta \right)$ 6:   ***End For*** 7: ***End For*** 8: $\rho_{ij;kL}=\frac{\rho_{ij;L}-\rho_{ik;L}\rho_{jk;L}}{\left(1-\rho_{ik;L}^{2}\right)\left(1-\rho_{jk;L}^{2}\right)}$


Three phenotypes were simulated by supplying different means for a small proportion of simulated metabolite blocks. A reference phenotype had $\mu_{k}=0$ for all k. The number of perturbed blocks in the comparator phenotypes was generated to follow a discrete uniform distribution, Unif(1,5). The perturbed block means were generated using a hierarchical model with $\mu_{ki}\sim N\left(\theta_{k},1\right)$ and $\theta_{k}\sim Exp\left(1/2\right)$. A simulated Bernoulli process with p=1/2 was employed to modulate the sign of $\theta_{k}$. The “realistic” scenario data was generated as above with the added data generation step of applying a nonlinear transformation to the empirical cumulative distribution function of the multivariate gaussian blocks to generate randomly-parameterized general gaussian distributions (GGD). The probability distribution function of a GGD with location parameter zero is defined as [35]:

(1) $$f_{x}\left(x\right)=\{\begin{array}{c} \phi \left(-\frac{1}{\kappa }\log1-\frac{\kappa x}{\alpha }\right)\;if\;\kappa \neq 0\\ \phi \left(x/\alpha \right)\;if\;\kappa =0 \end{array},$$

where ϕ is the standard Gaussian probability distribution function. In addition to introducing non-normal error distributions, a dirichlet-multinomial hierarchical model was used for simulating unbalanced phenotype distributions, Bernoulli processes were added to simulate biological and technical outliers, and an artificial lower limit of detection was introduced yielding a missing not at random (MNAR) mechanism. 

To evaluate the hypothesis that significance filtering prior to classifier construction would have an impact on the relative performances of the techniques evaluated, within each simulation study, we evaluated performance prior-to and post-significance filtering. Significance filtering was conducted by filtering on metabolites with significant pairwise *t*-tests between groups at a significance level of α=0.025 in the baseline scenarios and pairwise wilcoxon rank-sum tests in the realistic scenarios.

### 4.2. Classification Techniques

#### 4.2.1. Partial Least Squares-Discriminant Analysis (PLS-DA)

Partial least squares (for linear regression: PLS-R) has the following model formulation [36,37,38]:

(2) $$X=TP^{T}+E,\;Y=UQ^{T}+F.$$

In this formulation, E and F represent Gaussian noise, T and U are latent component matrices, and P and Q are the loading matrices that relate the latent components to the observed metabolite abundances X and the observed response variables Y. PLS algorithms seek to find weight vectors w and c such that ${\left[\mathrm{Cov}\left(t,u\right)\right]}^{2}={\left[\mathrm{Cov}\left(Xw,Yc\right)\right]}^{2}$ is maximized [39]. Specifically, the nonlinear iterative partial least squares algorithm (NIPALS) may be used to find w and c. The algorithm pseudocode is presented as in rosipal [40] below (Algorithm 2). Until convergence, repeat:

**Algorithm 2**1: **w = X^T^ u/(u^T^ u)** 2: **‖w‖→1** 3: **t = Xw** 4: **c=Y^T^ t/(t^T^ t)** 5: **‖c‖→1** 6: **u=Yc.**

Finally, p and q can be found by ordinary least squares (OLS) regression and X and Y are deflated. In our simulation studies and applications, we consider the first three score vectors, ${\left\{t_{i}\right\}}_{i=1}^{3}$. To utilize PLS for classification Y is defined as a binary indicator matrix of sample phenotypes and a discriminant analysis is conducted following regression.

#### 4.2.2. Sparse Partial Least Squares-Discriminant Analysis (sPLS-DA)

Lê Cao et al., [11] proposed a $l_{1}$ regularized version of PLS-DA to encourage sparsity in PLS modeling. In this section, we modify the description found in Lê Cao et al., [41] to maintain consistency. The objective of sPLS remains to find w and c such that ${\left[\mathrm{Cov}\left(Xw,Yc\right)\right]}^{2}$ is maximized, but now subject to penalization of the norm of w. To proceed, we introduce a result from höskuldsson [39], that w and c are the vectors that satisfy:

(3) $${\left[\mathrm{Cov}\left(t,u\right)\right]}^{2}={\left[\mathrm{Cov}\left(Xw,Yc\right)\right]}^{2}=\underset{f,g}{\max}{\left[\mathrm{Cov}\left(f,g\right)\right]}^{2},$$

given the singular value decomposition: $X^{T}Y=\underset{i}{\sum }a_{i}f_{i}g_{i}^{T}$. Consequently, the regularized optimization problem can be restated as:

(4) $$\underset{f_{h}g_{h}}{\min}||X_{h}^{T}Y_{h}-f_{h}g_{h}^{T}||+P_{\lambda }\left(f_{h}\right),$$

where h=1,2,…,H is the number of deflations. The penalization parameter λ was selected in each simulation study or real data analysis utilizing a grid search strategy (see Section 4.3).

#### 4.2.3. Support Vector Machines (SVM)

Support vector machines (SVM) are binary classifiers that seek to find hyperplanes that maximize the separation between classes. These hyperplanes may be linear in the original space of metabolite abundances (of dimension p) or in a higher dimensional space (of dimension p′) that allow for nonlinear boundaries in the original space [18]. A decision hyperplane for binary classification with ${\hat{y}}_{i}\in \left\{-1,1\right\}$ as phenotype indicators has the following form [13]:

(5) $${\hat{y}}_{i}=\mathrm{sign}\left(w^{T}\phi \left(x_{i}\right)+w_{0}\right),$$

where ϕ is an arbitrary real valued function and w is a vector of weights. This leads to the following optimization problem for M=1/||w|| [13,18]:

(6) $$\min_{w}\left|\left|w\right|\right|\;\mathrm{subject}\;\mathrm{to}y_{i}\left(w^{T}\phi \left(x_{i}\right)+w_{0}\right)\geq M\;\forall i.$$

As the optimization problem in (6) seeks to maximize the margin, M, that separates the phenotypes, SVM classifiers are often referred to as maximal margin classifiers. In the case that a hyperplane does not separate the observations by phenotype, then slack terms, $\xi_{i}$, are added allowing for a “soft” margin and yielding the optimization problem:

(7) $$\min_{w}\left|\left|w\right|\right|\;\mathrm{subject}\;\mathrm{to}y_{i}\left(w^{T}\phi \left(x_{i}\right)+w_{0}\right)\geq 1-\xi_{i}\;\forall i;\;\xi_{i}\geq 0;\;\sum^{\text{​}}\xi_{i}\leq c,$$

where c is a constant. The optimization problem is then solved by quadratic programming utilizing Lagrange multipliers. Conveniently, this optimization does not require explicit computation of the original data given new basis functions, that is $\phi \left(x_{i}\right)$, but rather the inner products $\phi \left(x_{i}\right),\phi \left(x_{i^{\prime }}\right)$ [13,18]. Consequently, nonlinear transformations are usually defined in terms of the kernel function determined by the inner product, $K\left(x,x^{\prime }\right)=\phi \left(x\right),\phi \left(x\prime \right)$. In each simulation study or real data analysis, Radial (Gaussian) kernels: $K\left(x,x^{\prime }\right)=\exp\left(-\gamma ||x-x^{\prime }{||}^{2}\right)$, were utilized with γ selected using a grid search strategy (see Section 4.3). As SVMs are binary classifiers, to employ SVMs for multi-phenotype discrimination, multiple classifiers need to be constructed and aggregated. In our analyses, we employ a “one-against-one” approach [42].

#### 4.2.4. Neural Networks (NNet)

In this analysis, we evaluated feedforward Neural Networks for classification. A feed forward network is a class of directed acyclic graphs loosely inspired by models of cognition in which metabolite abundances are conceptualized as stimuli and phenotype predictions are conceptualized as perceptions [13,21]. Topologically, a feedforward network consists of an input layer (allowing for the transfer of metabolite abundances), one or more hidden layers for processing and aggregation of signals from earlier layers, and an output layer with each phenotype represented by a node. Bias nodes may be incorporated to introduce signal independent of topologically antecedent layers. Given this topological representation, the general formula (output for each phenotype) with implicit bias terms is [21]:

(8) $$y_{g}=f_{O}\left(\underset{i\to k}{\sum }w_{ik}x_{i}+\underset{j\to k}{\sum }w_{jk}f_{h}\left(\underset{i\to j}{\sum }w_{ij}x_{i}\right)\right),$$

where $f_{O}$ and $f_{h}$ are continuous functions applied at output and hidden layer vertices, respectively; i→j represent directed edges between input layer vertices and hidden layer vertices; j→k represent directed edges between hidden layer vertices and output layer vertices; and i→k represent “skip-layer” transfers from input layer vertices directly to output layer vertices. In our analyses, we have utilized “Resilient Backpropagation” (RPROP) for training Neural Network classifiers [43]. In general, backpropagation algorithms iteratively use training observations to compute the output of a network (“forward pass”) followed by computation of the partial derivatives of the error function with respect to network weights (“backward pass”) for updating the weights by gradient descent [21,44]. Resilient backpropagation modifies the weight updating step to adaptively modulate the magnitude of weight updating based on the sign of the partial derivatives [43].

#### 4.2.5. Random Forests (RF)

A Random Forest (RF) classifier can be conceptualized as an ensemble of M classification trees each constructed utilizing a bootstrap sample from the original data. The process of constructing individual classification trees proceeds by recursive binary splits (splitting a parent node into two daughter nodes) selected from a restricted subset of random variables (metabolites) and cutpoints [17,18]. Specifically, at each iteration, a set of candidate regions:

(9) $$ℛ=\left\{R_{L}\left(X_{j},s\right),R_{R}\left(X_{j},s\right)\right\}=\left\{\left\{X_{j}|X_{j}\leq s\right\},\left\{X_{j}|X_{j}\rangle s\right\}\right\}$$

is generated following the selection of a set of random variables (metabolites) sampled with replacement from the bootstrapped data. After generating the regions, the empirical phenotype distribution is computed over each region R, that is:

(10) $${\hat{\pi }}_{Rg}=\frac{1}{N\left(R\right)}\sum_{i:X_{ij}\in R}I\left(y_{i}=g\right),$$

for each phenotype g. For each region, a phenotype is then ascribed: ${\hat{y}}_{i}=\arg\max_{g}{\hat{\pi }}_{Rg}.$

$X_{j}$ and s are then chosen to minimze a measure of node impurity—in our case, the misclassification error: $1-\frac{1}{N\left(R\right)}\underset{i:X_{ij}\in R}{\sum }I\left({\hat{y}}_{i}=y_{i}\right)$. Once $X_{j}$ and s have been selected, the current parent node is split into the daughter nodes satisfying $\left\{x_{i}:\;x_{ij}\leq s\right\}$ or $\left\{x_{i}:x_{ij}>s\right\}$. When generating an ensemble of individual classification trees, the correlation between individual trees estimated from the bootstrapped samples is reduced by enforcing a random subspaces constraint [20], considering at each binary split only a randomly drawn subset of variables (metabolites). Once an ensemble of trees has been aggregated as a Random Forest, predicted phenotype probabilities can be determined by aggregating individual tree predictions.

#### 4.2.6. Naïve Bayes (NB)

Naïve Bayes classifiers are derived from a straightforward application of Bayes’ theorem to multiclass classification [13,18], that is:

(11) $$P\left(c_{g}|x\right)=\frac{P\left(c_{g}\right)P\left(x|c_{g}\right)}{P\left(x\right)}.$$

Noting that P(x) is independent of $c_{g}$, the phenotype label, the posterior distribution of phenotype labels is then proportional to the numerator of Label (11) only. Given the “naïve” assumption that the metabolite abundances are independent the posterior probability is then:

(12) $$P\left(c_{g}|x\right)\propto P\left(c_{g}\right)\prod_{j=1}^{p}P\left(x_{j}|c_{g}\right).$$

A Gaussian distribution is then assumed for each metabolite conditioned on phenotype, that is $P\left(x_{j}|c_{g}\right)\sim N\left(\mu_{j},\sigma_{j}^{2}\right)$, and the Gaussian distribution parameters are estimated via maximum likelihood estimation.

#### 4.2.7. *k*-Nearest Neighbors (*k*-NN)

*k*-Nearest Neighbors classifiers can also be derived from an application of Bayes’ theorem [13]. Given a set of training samples $\left\{x_{i},y_{i}\right\}$ where $x_{i}$ represents metabolite abundances and $y_{i}=c$ represents the sample phenotype, phenotype probabilities for a new sample $x_{i^{\prime }}$ can be estimated using a neighborhood $N_{k}\left(x_{i^{\prime }}\right)$ of the closest training samples with respect to a distance metric $d\left(x,x^{\prime }\right)$ such as Euclidean distance. Representing number of samples within $N_{k}\left(x_{i^{\prime }}\right)$ with phenotype label $c_{g}$ as $N_{c_{g}}$ and the total number of samples within $N_{k}\left(x_{i^{\prime }}\right)$ as N, the posterior probabilities of phenotype label are then:

(13) $$P\left(c_{g}|x_{i^{\prime }}\right)=\frac{P\left(c_{g}\right)P\left(x_{i^{\prime }}|c_{g}\right)}{P\left(x_{i^{\prime }}\right)}=\frac{N_{c_{g}}}{N}.$$

### 4.3. Parameter Selection

Each of the classification techniques evaluated in the present study represent families of classifiers whose members are uniquely determined by algorithm parameters. Consequently, we sought to minimize the probability that an observed relative difference in classifier performance was due to sub-optimal parameter selection for one or more techniques. During the course of each simulation study (prior-to and post-significance filtering), parameter selection was conducted by minimizing expected cross-entropy loss estimated by cross-validation and smoothed over a parameter grid using kernel smoothing. For reproducibility, the relevant fixed and cross-validation selected algroithm parameters used in defining the classifiers are shown in Table 4.

### 4.4. Evaluation of Classifier Performance

Classifier performance was evaluated by computation of the empirical risk (error) associated with two different loss functions [44]. Defining a phenotype prediction ${\hat{y}}_{i}=arg{max}_{g}{\hat{\pi }}_{ig}$ from a classifier, a 0–1 loss function is: $L\left({\hat{y}}_{i},y_{i}\right)=I\left({\hat{y}}_{i}\neq y_{i}\right)$, with associated empirical risk (the misclassification rate): $1/N\overset{N}{\underset{i=1}{\sum }}I\left({\hat{y}}_{i}\neq y_{i}\right)$. Cross-entropy loss is defined as: $-\overset{G}{\underset{g=1}{\sum }}I\left(y_{i}=g\right)log{\hat{\pi }}_{ig}$ with empirical error: $-1/N\overset{N}{\underset{i=1}{\sum }}\overset{G}{\underset{g=1}{\sum }}I\left(y_{i}=g\right)log{\hat{\pi }}_{ig}$. While the misclassification rate measures the frequency of a classifier incorrectly classifying observations, the empirical cross-entropy error measures the average amount of extra information required to represent the true phenotypes with the predicted phenotypes. Consequently, the empirical cross-entropy error provides a measure of how well the predicted phenotypes “match” the true phenotypes. The distinction between these loss functions can be observed with the following case. Given a binary classification task, a misclassified observation with a predicted phenotype probability of 49% incurs less cross-entropy loss than a predicted phenotype probability of 0.1%. Given a 0–1 loss function, the computed loss would be the same for a misclassified observation with a predicted phenotype probability of 49% as a predicted phenotype probability of 0.1%.

### 4.5. Clinical Datasets

In addition to evaluation of classifier performance via simulation studies, classifier performance was evaluated over two clinical datasets. In the first, DeFilippis et al., [6] employed an untargeted approach for determining a plasma signature that differentiates between thrombotic myocardial infarction (MI), non-thrombotic MI, and stable coronary artery disease (CAD). Thrombotic MI is characterized by atherosclerotic plaque rupture/disruption that leads to the formation of a thrombus and the obstruction of a coronary artery [45] while non-thrombotic MI occur secondary to other causes such as blood supply demand mismatch during tachyarrhythmias, coronary artery spasm or low blood oxygen levels. Plasma samples from 23 subjects presenting with acute MI and 15 subjects with stable coronary artery disease undergoing cardiac catheterization were analyzed. Of the 23 acute MI subjects, 11 were adjudicated to be thrombotic MI and 12 were adjudicated to be non-thrombotic MI utilizing a strict criteria. 1,032 metabolites were detected and quantified by gas chromatography mass spectrometry (GC-MS with electron ionization), and ultra performance liquid chromatography mass spectrometry (UPLC-MS with electrospray ionization) in both positive and negative ion modes. Given the limited sample size, we employed a cross-validation approach to measuring classifier performance. In the second dataset, Fahrman et al. [46] sought to determine plasma or serum based biomarkers that could be used to detect adenocarcinoma lung cancer with better specificity than existing methods such as low-dose computed tomography. The researchers developed two case-control cohorts for the purpose of discovering and validating biomarkers of adenocarcinoma lung cancer. Untargeted gas chromatography time-of-flight mass spectrometry with electron ionization was used to determine metabolic abundances in both the discovery and validation cohorts. In our analysis of classifier performance, we utilized the plasma sample metabolite abundances from the second cohort and employed a train-test approach. In the second cohort, abundances of 413 metabolites were reported. In the final dataset, Zhang [47] conducted a metabolomics analysis of serum from healthy subjects with different NOS1AP (Nitric Oxide Synthase 1 Adaptor Protein) rs12742393 polymorphisms. In this serurm from AA, AC, CC genotypes were examined by GC-TOF-MS and UPLC-QTOF-MS. Error was quantified over the adenocarcinoma dataset and the NOS1AP dataset using withheld test sets of 1/3 of the total observations. Over the acute MI dataset, error was estimated using repeated double cross-validation [48]. 

### 4.6. Statistical Software

The simulation studies and analyses over real datasets were conducted in the R environment [49] and made use of functions from the following packages: clusterGeneration [50], class [51], randomForest [52] , e1071 [53], neuralnet [54], caret [55], cvTools [56], dplyr [57], and tidyr [58]. 

## 5. Conclusions

The analysis reported supports a few conclusions regarding classifier accuracy for application in untargeted metabolomics. In the most realistic simulation studies that incorporated non-normal error distributions, unbalanced phenotype allocation, outliers, missing values, and dimension reduction, classifier performance (least to greatest error) was ranked as follows: SVM, Random Forest, Naïve Bayes, sPLS-DA, Neural Networks, PLS-DA and *k*-NN classifiers. When non-normal error distributions were introduced, the performance of PLS-DA and *k*-NN classifiers deteriorated further relative to the remaining techniques. Over the real datasets, a trend of better performance of SVM and Random Forest classifier performance was observed. Finally, this work demonstrates that relative classifier performance is not invariant given choice of loss function. 

## Figures and Tables

**Table 1:** Misclassification rate (%) observed by technique and by significance filtering status (pre- vs. post**-**) throughout the 1000 baseline simulation studies and 1000 realistic simulation studies. The lowest median misclassification rate observed over each scenario type is shown in bold face.

Method	Baseline Pre-		Baseline Post-		Realistic Pre-		Realistic Post-	
	Mean ± SD	Median ± IQR	Mean ± SD	Median ± IQR	Mean ± SD	Median ± IQR	Mean ± SD	Median ± IQR
PLS-DA	15.0 ± 15.5	10.0 ± 28.3	13.1 ± 15.6	5.0 ± 25.0	32.2 ± 17.4	30.0 ± 26.7	25.0 ± 15.7	23.3 ± 24.6
sPLS-DA	13.1 ± 15.3	**5.0 ± 25.0**	12.1 ± 15.1	3.3 ± 23.3	19.7 ± 15.2	15.0 ± 23.3	22.0 ± 15.0	20.0 ± 21.7
SVM	23.2 ± 24.6	13.3 ± 41.7	10.4 ± 14.3	**1.7 ± 19.6**	22.8 ± 18.3	16.7 ± 26.7	13.3 ± 12.5	**8.3 ± 13.3**
NNet	22.0 ± 15.6	20.0 ± 25.0	15.9 ± 13.8	11.7 ± 21.7	29.9 ± 15.2	28.3 ± 21.7	23.3 ± 14.3	21.7 ± 21.7
RF	15.0 ± 14.8	10.0 ± 23.3	13.5 ± 14.4	7.5 ± 21.7	17.5 ± 16.0	**11.7 ± 21.7**	15.5 ± 15.0	10.0 ± 18.3
*k*-NN	20.2 ± 17.3	20.0 ± 28.3	21.9 ± 16.4	20.0 ± 25.0	41.3 ± 18.8	41.7 ± 26.7	41.6 ± 17.7	41.7 ± 26.7
NB	14.0 ± 15.3	8.3 ± 25.0	11.1 ± 14.6	1.8 ± 21.7	32.1 ± 18.2	30.0 ± 28.3	19.1 ± 14.8	15.0 ± 21.7

PLS-DA: Partial Least Squares-Discriminant Analysis; sPLS-DA: Sparse PLS-DA; SVM: Support Vector Machines; NNet: Artificial Neural Network; RF: Random Forest; *k*-NN: *k*-Nearest Neighbors; NB: Naïve Bayes

**Table 2:** Cross-entropy loss observed by technique and by significance filtering status (pre- vs. post-) throughout the 1000 baseline simulation studies and 1000 realistic simulation studies. The lowest median cross-entropy loss observed over each scenario type is shown in bold face.

Method	Baseline Pre-		Baseline Post-		Realistic Pre-		Realistic Post-	
	Mean ± SD	Median ± IQR	Mean ± SD	Median ± IQR	Mean ± SD	Median ± IQR	Mean ± SD	Median ± IQR
PLS-DA	1.18 ± 0.16	1.17 ± 0.23	1.04 ± 0.18	0.99 ± 0.30	1.51 ± 0.14	1.51 ± 0.19	1.50 ± 0.16	1.50 ± 0.20
sPLS-DA	1.03 ± 0.18	0.98 ± 0.30	1.02 ± 0.19	0.96 ± 0.29	1.49 ± 0.16	**1.48 ± 0.20**	1.50 ± 0.16	**1.49 ± 0.20**
SVM	0.64 ± 0.45	0.70 ± 0.61	0.42 ± 0.40	0.21 ± 0.56	1.95 ± 0.71	1.83 ± 0.95	1.90 ± 0.61	1.81 ± 0.77
NNet	1.12 ± 0.20	1.09 ± 0.32	1.03 ± 0.18	0.97 ± 0.29	1.53 ± 0.17	1.54 ± 0.22	1.51 ± 0.18	1.52 ± 0.22
RF	0.58 ± 0.36	**0.55 ± 0.52**	0.55 ± 0.34	0.51 ± 0.50	6.61 ± 17.57	1.66 ± 0.92	6.37 ± 16.38	1.65 ± 0.92
*k*-NN	54.8 ± 56.4	44.9 ± 54.3	60.8 ± 52.9	50.6 ± 46.1	345.0 ± 181.6	326.5 ± 257.2	343.7 ± 171.9	328.9 ± 244.3
NB	3.12 ± 3.86	1.20 ± 5.56	0.94 ± 1.27	**0.11 ± 1.85**	129.0 ± 113.4	94.4 ± 119.5	96.9 ± 92.2	65.7 ± 94.3

PLS-DA: Partial Least Squares-Discriminant Analysis; sPLS-DA: Sparse PLS-DA; SVM: Support Vector Machines; NNet: Artificial Neural Network; RF: Random Forest; k-NN: k-Nearest Neighbors; NB: Naïve Bayes

**Table 3:** Misclassification rate and cross-entropy loss observed over real datasets. Pre- represents pre-significance filtering while post- represents post-significance filtering. Lowest error is shown in bold face.

Dataset	Technique	Misclassification (%)		Cross-Entropy Loss	
		Pre-	Post-	Pre-	Post-
Adenocarcinoma	PLS-DA	**17.9**	**7.1**	0.78	0.68
	sPLS-DA	32.1	14.3	0.83	0.72
	RF	**17.9**	14.3	**0.68**	0.57
	SVM	21.4	10.7	0.78	**0.53**
	NNet	21.4	28.6	0.77	0.86
	*k*-NN	28.6	14.3	61.2	30.8
	NB	**17.9**	10.7	4.85	2.56
Acute MI	PLS-DA	47.4	42.1	1.41	1.28
	sPLS-DA	47.4	15.8	1.43	1.35
	RF	**22.1**	**7.9**	**1.08**	0.76
	SVM	55.3	13.2	1.89	**0.65**
	NNet	47.4	31.6	1.47	1.16
	*k*-NN	44.7	39.5	95.3	106.4
	NB	42.1	15.8	164.0	20.3
NOS1AP	PLS-DA	22.9	6.3	1.14	0.93
Variants	sPLS-DA	**2.1**	6.3	0.98	0.93
	RF	6.3	**4.2**	**0.27**	**0.21**
	SVM	12.5	6.3	0.50	0.26
	NNet	16.7	6.3	1.08	0.86
	*k*-NN	41.7	8.3	88.8	17.8
	NB	12.5	6.3	4.14	1.75

**Table 4:** Simulation study parameters.

Technique	Parameter	Type	Value/Search Grid
PLS-DA	Number of components	Optimized	[1, 2, ..., 15]
Sparse PLS-DA	Number of components	Optimized	[1, 2, ..., 15]
	Regularization (λ)	Optimized	[0.1, ..., 0.9] by 0.1
Random Forest	Ensemble size	Fixed	1000
	Random subspace size	Optimized	[5, ..., *p*] of length 25
SVM	Kernel	Fixed	Gaussian
	Bandwidth (γ)	Optimized	10^[−5, ..., −1] of length 1000 ^†^; 10^[−2, ..., 0] of length 1000 ^‡^
Neural Network	Number of hidden layers	Optimized	1 or 2
	Number of hidden nodes	Optimized	[15, ..., 100] by 5
	Activation function	Fixed	Logistic
	Learning function	Fixed	Resilient Backpropagation
	Error function	Fixed	Cross-entropy Loss
*k*-NN	Number of neighbors	Optimized	[1, 2, ..., 20]

^†^ Prior-to significance filtering. ^‡^ Post-significance filtering.

## Supplementary Materials

R scripts for conducting the simulation studies are publicly available via GitHub (http://github.com/trainorp/MetabClass).
